# Immunity to Avian Leukosis Virus: Where Are We Now and What Should We Do?

**DOI:** 10.3389/fimmu.2016.00624

**Published:** 2016-12-21

**Authors:** Min Feng, Xiquan Zhang

**Affiliations:** ^1^Department of Animal Genetics, Breeding and Reproduction, College of Animal Science, South China Agricultural University, Guangzhou, China; ^2^Guangdong Provincial Key Lab of Agro-Animal Genomics and Molecular Breeding and Key Lab of Chicken Genetics, Breeding and Reproduction, Ministry of Agriculture, Guangzhou, China

**Keywords:** ALV, innate immunity, adaptive immunity, chicken, retrovirus

## Abstract

Avian leukosis virus (ALV) is an avian oncogenic retrovirus causing enormous economic losses in the global poultry industry. Although ALV-related research has lasted for more than a century, there are no vaccines to protect chickens from ALV infection. The interaction between chickens and ALV remains not fully understood especially with regard to the host immunity. The current review provides an overview of our current knowledge of innate and adaptive immunity induced by ALV infection. More importantly, we have pointed out the unknown area involved in ALV-related studies, which is worthy of our serious exploring in future.

## Introduction

Avian leukosis virus (ALV) is a notorious retrovirus causing neoplastic disease, immunosuppression, and other production problems. In chickens, the ALV are divided into six subgroups, including A, B, C, D, E, and J, based on their viral envelope glycoproteins responsible for viral interference patterns, virus neutralization, and host range (1). It is glorious that ALV-related studies provided Nobel laureates in 1966, 1975, and 1989 (1). Nowadays, ALV was almost eradicated in the western world (2, 3), but in China ALVs still persist in many bird species (4–8). Moreover, because Chinese poultry industry is less organized, especially among the local breeds of chickens, the ALV will exist for a long time and related studies would keep on in China.

Historically, the primary aims of ALV studies were concerned with the virus itself. This included elucidating mechanisms of tumorigenesis, viral transmission, virus isolation, viral replication, pathogenesis, and molecular biology. However, studies concerning the innate and adaptive immune responses to ALV have been neglected. Some reported studies are just limited on immunologic tolerance and immunosuppression induced by ALV (9, 10).

The purpose of this review is to provide an overview of progress in immunity against ALV, broaden the scope in new areas that are under active investigation.

## Immunity Against ALV

### Innate Immunity

The innate immune response provides the first line of defense against invading viruses and plays a key role in the subsequent activation of antiviral responses. During viral infection, virus pathogen-associated molecular patterns (PAMPs) are recognized by pathogen recognition receptors (PRRs) that include toll-like receptors (TLRs) (11), retinoic acid-inducible gene I (RIG-I)-like receptors (RLRs), NOD-like receptors (12), interferon-γ-inducible protein 16 (IFI16), and cyclic GMP-AMP synthase (cGAS) (13).

Virus recognition activates signaling pathways that lead to interferon (IFN) production as well as the activation of inflammatory cytokines and chemokines (12). These immune factors recruit and activate innate immune cells including macrophages, dendritic cells (DCs), and natural killer (NK) cells that can control virus spread and activate and modulate the adaptive immune response (14). Moreover, hundreds of interferon-stimulated genes (ISGs) are induced through the JAK-STAT pathway and interact directly with viruses (15). Here, we will comprehensively discuss the interactions between ALV and host innate immunity regarding to the PRRs recognition, cytokine production, ISGs expression, and innate immune cells activation.

### ALV Sensing

As single-stranded RNA retrovirus, ALV, like HIV, should theoretically be recognized by PRRs such as TLRs, RLRs, IFI16, and cGAS (13, 16–18). However, which specific innate sensors response to ALV is still elusive.

In DCs, TLR (1–4) expression changed significantly after ALV-J infection (19). However, the inclusion of lipopolysaccharide and interleukin-4 (IL-4) as pretreatments in these studies hampered the determination of which TLR was responsible (19). On the other hand, melanoma differentiation-associated gene 5 (MDA5) was found to have differential expression in ALV-J infected chickens identified through the use of transcriptome analysis with hybridization arrays and RNA-Seq (20, 21). In our laboratory, we demonstrated that ALV-J infection significantly increased TLR7 expression in chicks followed by MDA5 when the infection progressed to tumorigenesis (22). As a model, HIV-1 recognition by TLR7 requires only attachment and endocytosis, independent of retroviral replication (23). TLR-7 expression increased in ALV-J infected chicks at 1-day post-hatch and suggested that ALV-J was recognized by chicken TLR7 during the initial infection. RIG-I is a cytoplasmic sensor for HIV genomic RNA (23). Chickens lack RIG-I but MDA5 can compensate in immune activation (24). Our results showed that MDA5 expression was induced in the tumorigenesis phase, and we speculated that MDA5 was the primary sensing PRR during later infection stages (22).

In future, it is necessary to further verify that TLR7 and MDA5 are specific sensors of ALV *via* more experiments. Besides, other PRRs involved in ALV infection need be explored and identified.

### Cytokine Production

The chicken spleen plays a dominant role in the generation of immune responses due to the absence of well-developed lymph nodes (25). This organ also functions in innate immune responses to ALV-J infection (26). In 1-day-old ALV-J-infected chicks, we could not detect any significant expression of IL-6, IL-10, IL-1β, or IFN-β in spleens from 1-day postinfection (dpi) to 7 dpi (22). In a similar study with ALV-J and 1-day-old chicks, IL-6, IL-18, IFN-α, and IFN-γ did not significantly change from 1 to 7 dpi, but they were significantly increased in spleens 9–12 dpi. The cytokine levels then sharply declined at 15 dpi when the ALV-J load reached its peak (26). Apparently, ALV-J does not induce an obvious antiviral innate immune response in 1-week-old chicks, and this helps to explain why ALV transmission primarily occurs at hatching or in the first week of life (27).

In the late stages of ALV-J infection, IL-6, IL-1β, IL-10, and IFN-β protein levels were significantly increased in the clinical infected chickens (22). In infected specific-pathogen-free chickens, IL-2 and IL-10 mRNA levels were significantly increased (28). IL-10 is a most important anti-inflammatory cytokine with immunosuppressive effects (29). High level of IL-10 (29) or large amounts of ALV-J might cause immunosuppression in chickens (26). In addition, these results suggest that IFN and interleukin play a role in the interaction of host innate immune system with ALV-J infection. We had previously determined that DF-1 (chicken embryo fibroblast) cells pretreated with recombinant chicken IFN-α were able to inhibit ALV-A/B/J replication (28). This study confirmed the importance of IFN in innate immunity against ALVs *in vitro*.

There have been few studies identifying specific inflammatory pathways in ALV-chicken interactions. However, a caspase-1-mediated inflammatory response could be triggered by ALV-J infection in chick livers (30). Caspase-1 expression combined with adaptor NLRP3 enabled IL-1β and IL-18 increases at 5 or 7 dpi (30). NLRP3 is an important initiator protein of the inflammasome, a multicomponent complex that activates caspase-1 and results in IL-1β and IL-18 secretion (31). However, this is the extent of this type of data but indicates that further research would yield fruitful results.

### IFN-Stimulated Gene Induction

Many viruses trigger the IFN system that leads to the transcription of hundreds of ISGs. These genes exert antiviral effector functions, many of which are still not fully understood (15). In chickens, ISGs are not generally well described with the exception of the chicken *ZAP* and *viperin* genes (32, 33).

Avian leukosis virus-A/B/J infections increase the promoter activity of chicken interferon regulatory factors 3 (IRF3) [more similar to IRF7 (34)] (28). However, there are still no published reports on the activation of transcription factors such as IRF3, NF-κB, and those in the JAK-STAT pathway. Similarly, the identity of ISGs that directly act against ALV has only recently been reported.

*In vivo* studies demonstrated that *ISG12-1, ISG12-2, OASL*, and *Mx* increased in the chicken bursa of Fabricius at the 18th day of embryonation, and in 10- and 30-day-old with ALV-J infection (20). However, during the late stages of ALV-J infection or in the presence of a tumor, *ISG12-1, ISG12-2, Mx, ZAP, IRF1*, and *STAT1* were significantly decreased or remained unchanged in chicken spleens (21, 22). This suggests that ALV may escape innate immunity result by decreasing some ISGs expression of during late infection stages (21, 22).

During ALV-J infection, miR-23b targeted *IRF1* and down-regulated IFN-β expression, further promoting ALV-J replication (21). Interestingly, chicken biliary exosomes were found to contain *ZAP* and these inhibited ALV-J replication *in vitro* (35). Chicken *ZAP* is expressed in response to H5N1 and IBDV infections (32), but whether chicken *ZAP* is the key factor that inhibited ALV-J replication requires further study. It is important to identify and verify additional chicken ISGs to broaden our understanding of innate immune responses to develop protective strategies against ALV infections in chickens.

### Innate Immune Cells

Virus sensing by PRRs leads to the immune activation of infected and accessory cells, accompanied by cytokine and chemokine production. The activation of innate immune cells may be a consecutive process, starting with macrophages and DCs and progressing to NK cells (13).

### Macrophages

The macrophage is the component of the first line of immune defense against pathogens. It possesses a wide range of functions including cytokine and chemokine secretion, phagocytosis, production of nitric oxide, and antigen presentation (36, 37). Several years ago, it was found that chicken macrophages were susceptible to ALV-B/C, whereas ALV-A/D was excluded. These viruses could persist in macrophages for long periods (38, 39). However, the immunologic function of the macrophage-ALV interaction has not been followed up.

Recently, we determined that chicken primary monocyte-derived macrophages (MDM) were susceptible to ALV-J (40). ALV-J strain SCAU-HN06 (41) rapidly increased the expression of *Mx, ISG12-1*, IL-1β, IL-6, and IFN-β in MDM at early infection stages, but *Mx, ISG12-1*, and IL-10 expression decreased sharply at 36 h postinfection (40). This result indicated that ALV-J most likely escaped the innate immune response in chicken macrophages. Retroviruses have the ability to evade immune defense system and establish long-term persistence in the infected hosts. Macrophages play critical roles in HIV infection and can be a viral reservoir (42). We speculate that ALV-J also evades the innate immune response and establishes latent infections in chicken macrophages.

### Dendritic Cells

As the most important professional antigen-presenting cells, DCs have a key role in the initiation and control of immunity (43). An *ex vivo* study demonstrated that ALV-J could infect bone marrow-derived DCs (BM-DCs) during the early stages of differentiation and trigger apoptosis (44). Further studies showed that ALV-J inhibits the differentiation and maturation of BM-DCs and alters cytokine expression, causing aberrant antigen presentation and an altered immune response (19).

As a central regulator of innate and adaptive immunity, DCs can stimulate T cells, antigen presentation, and secrete cytokines and chemokines (45, 46). In chickens, DCs-related research was initiated late because reproducible methods for culturing and characterizing this cell were only established in 2010 (47). The study on chicken DCs with ALV-J infection was still in the start stage, future studies are expected to unravel functions of chicken DCs.

### Natural Killer Cells

Unfortunately, we only found one paper related to the interaction between NK cells and ALV infection (48). From an immunosuppression standpoint, this study indicated that ALV-J-infected chicken NK cells had a lower killing activity than the NK cells of the uninfected controls (48). This is a promising start and we await further work.

Natural killer cells play an important role in host defense and tumor surveillance, ending in target cell death and chemokine and cytokine secretion (49). In addition, NK cells have a key role in immune regulation. NK cells can regulate T cell and DC functions in mouse models of viral infection (50, 51). Given that ALV is tumorigenic and NK cells are central innate immune effectors, we believe that further exploration into NK cells and ALV interactions is worthwhile. This is especially important in terms of immune regulatory functions and tumor immunity.

Chicken macrophages and DCs can be directly infected by ALV and induce innate immunity (40, 44). However, we still have no clear knowledge of the regulation of the global innate immune response to ALV infection and ALV evasion of the host innate immune response. It is necessary to define the mechanisms of innate immune control in ALV infection to understand the virus-host relationship more deeply. This could result in a major contribution to ALV vaccine development by providing effective adjuvants that target innate immunity.

### Adaptive Immune Responses

#### Humoral Immunity

Antibody responses to ALV are complex. Infection with ALV results in three classical infection profiles including (1) V+A− (viremia, no neutralizing antibody); (2) V+A+ (viremia, with neutralizing antibody); and (3) V−A+ (no viremia, with neutralizing antibody) (52, 53).

Congenital infection of chickens has been regarded as a classical model of immunologic tolerance that is demonstrated at the humoral level (10). Maternal antibodies against ALV-A influence the development of neutralizing antibody, viremia, and virus shedding (54). In general, an *in ovo* ALV infection results in persistent viremia lacking neutralizing antibody, and post-hatch ALV infection could potentially lead to clear the viruses by neutralizing antibody (55). Chickens infected with ALV-A after hatch often develop a transient viremia followed by an efficient neutralizing antibody response that is able to prevent viremia reappearance (52). However, high levels of ALV-J viremia can persist in the presence or absence of neutralizing antibody during the first 2 weeks post-hatch ALV-J infection (27, 56). This phenomenon suggests that an anti-ALV vaccine should be feasible.

However, ALV is a retrovirus, like HIV-1, has an unstable genome especially concerning mutations in envelope glycoproteins (57, 58). Developing a vaccine to induce effective neutralization antibody for ALV prevention represents a great challenge. In addition, neutralization antibody may not be sufficient to counter variants (55). Despite this, numerous anti-ALV vaccines were still developed. The specific details on developed ALV vaccine could be found in Table 1.

**Table 1:** **Avian leukosis virus (ALV) vaccine trials**.

ALV strain	Vaccine components	Adjuvant	Results summary	Immunological target	Reference
J	Recombinant ALV-J gp85 protein	Liposomes	High antibody levels; 58.3% (inoculation once) and 83.3% (inoculation twice) protection ratios	Neutralizing antibody	(59)
J	Recombinant ALV-J gp85 protein	Cytosine-phosphate-guanine oligodeoxynucleotide (CpG-ODN)	Inducing breeder hens to produce effective maternal antibody that protected the hatched chickens against early ALV-J infection (70% protection ratios)	Neutralizing antibody	(60)
J	Recombinant chimeric multi-epitope protein X	Freund’s adjuvant	80% protection ratios	Neutralizing antibody and cellular responses	(61)
J	DNA vaccine with chimeric multi-epitope DNA	Freund’s adjuvant	70% protection ratios	Neutralizing antibody and cellular responses	(62)
A	Recombinant ALV-A gp85 protein	CpG-ODN	Inducing the breeder hens to produce better neutralizing antibody responses and protect 80% of their offspring chickens against early infection	Neutralizing antibody	(69)
B	Inactivated ALV-B vaccine	Oil	Inducing antibody reaction to ALV-B and providing maternal antibodies to 1-day-old chickens against early infection of ALV-B	Neutralizing antibody	(68)

The recombinant ALV-J gp85 protein vaccine provided with either a liposomal or a cytosine-phosphate-guanine oligodeoxynucleotide (CpG-ODN) adjuvant did provide partial protection and elicited high antibody titers (59, 60). More interestingly, there has been a multi-epitope subunit vaccine developed that induced significant humoral and cellular immune responses in chickens against ALV-J infection (61). In a similar manner, a chimeric multi-epitope-based DNA vaccine can elicit higher antibody titers and cellular responses against an ALV-J challenge in chickens (62). The chimeric multi-epitope gene of ALV-J including 4 multi-epitope concentrated fragments (gag, pol, gp85, and gp37) encodes recombinant chimeric multi-epitope protein X containing both immunodominant B and T epitope (63). The vaccine with single antigen may lead to less immunogenicity and limited protection (64). However, multi-epitope based vaccines could increase immunogenicity and enhance immune responses due to containing epitopes of different target genes (65, 66). Thanks to cellular immune responses can complement antibody-mediated protection (67), we think the vaccine which provides antibody protection and induces cellular immune should be developed preferentially.

An inactivated ALV-B vaccine has been developed that could induce high antibody titers that protect from experimental ALV-B infections in chickens (68). An ALV-A gp85 protein subunit vaccine could induce neutralizing antibody when given with a CpG-ODN adjuvant to breeder hens. This protected 80% of their offspring chickens against early infections (69).

According to the published data, we found that the protection ratio of each developed vaccine is higher. However, we still doubt the vaccines’ effect because of fewer experimental animals and shorter monitoring time. In fact, none of the vaccines has a clinical application in chicken farms.

But nevertheless, the vaccine is still a kind of technical reserve to control ALV. What’s more, the development of an ALV vaccine may also serve as a model for HIV vaccine development. Therefore, exploring this avenue is worthy of our serious consideration. Recently, new B-cell epitopes in the P27 or gp85 proteins have been discovered, which holds promise as novel vaccine agents (70–72).

#### Cellular Immunity

There are limited studies concerning cellular immunity in ALV infections. A role for cellular immunity was correlated with immunosuppression of T-cell function in ALV-A infected chickens (73). Cytotoxic T lymphocytes were shown to play a role in the susceptibility or resistance of the various MHC-I haplotype chicken lines to ALV-A infection (74). However, the pathogenesis of immunosuppression caused by ALV-J may be associated with both B and T cells (9).

A key period for developing immunosuppression to ALV-J infection was identified as 3–4 weeks postinfection (9). At this stage, CD4^+^ T-cell numbers were significantly reduced and the CD8^+^ T-cell lymphocyte population increased in the spleen (9). Coincidentally, an untreated HIV infection is characterized by progressive CD4^+^ T cell depletion and CD8^+^ T cell expansion (75). Therefore, CD4^+^ T cells may be a primary target for ALV-J with CD8^+^ T cells playing an important role in host immunity.

Avian leukosis virus-J infection inhibits blood and splenic T lymphocyte proliferation and cytotoxicity in broilers. This effect can be enhanced by co-infection with reticuloendotheliosis virus (REV) (76). Interestingly, the joint application of Taishan *Pinus massoniana* pollen polysaccharides and propolis improved immune system effectiveness that included raising CD4^+^ and CD8^+^ T-cell counts as well as IL-2 and IFN-γ secretion in immunosuppressed chickens caused by ALV-J co-infection with REV (77). A separate study found chicken biliary exosomes significantly inhibited ALV-J replication and promoted proliferation of CD4^+^ (especially CD4^+^CD8^–^cells) as well as CD8^+^ T cells (35). However, whether the cellular immunity changed by biliary exosomes plays a dominant role by inhibiting ALV-J replication remains unknown.

A potential vaccine for ALV-J has been reported to induce significant increases in CD4^+^ and CD8^+^ T cells as well as IL-4 and IFN-γ levels in immunized chickens (61). Unfortunately, there have been few studies involved in the specific functions of T cells against ALV in the all cases described above.

We are sure that cellular immunity plays a critical role in ALV infection, but know little about the ALV-specific response. A full understanding of the ALV-specific cellular immune response is necessary to develop effective vaccines. Perhaps more importantly, this type of work can serve as a reference for HIV prevention and treatment with prophylactic vaccines or immunotherapies. Indeed, escape mutations and retrovirus latent infections are the main barriers in this effort.

## Future Research

Many interesting scientific questions about ALV remain unresolved. Besides, cancer and AIDS are still great threats to human health. ALV studies might make a major contribution to conquer cancer and HIV as a research model. There are no vaccines to prevent HIV infection and many high-budget vaccine programs for HIV have continuously failed (78, 79). Developing ALV vaccine studies have a realistic significance and greater knowledge of immune system-ALV interactions may provide great insights into human retroviral diseases.

**Figure 1:**
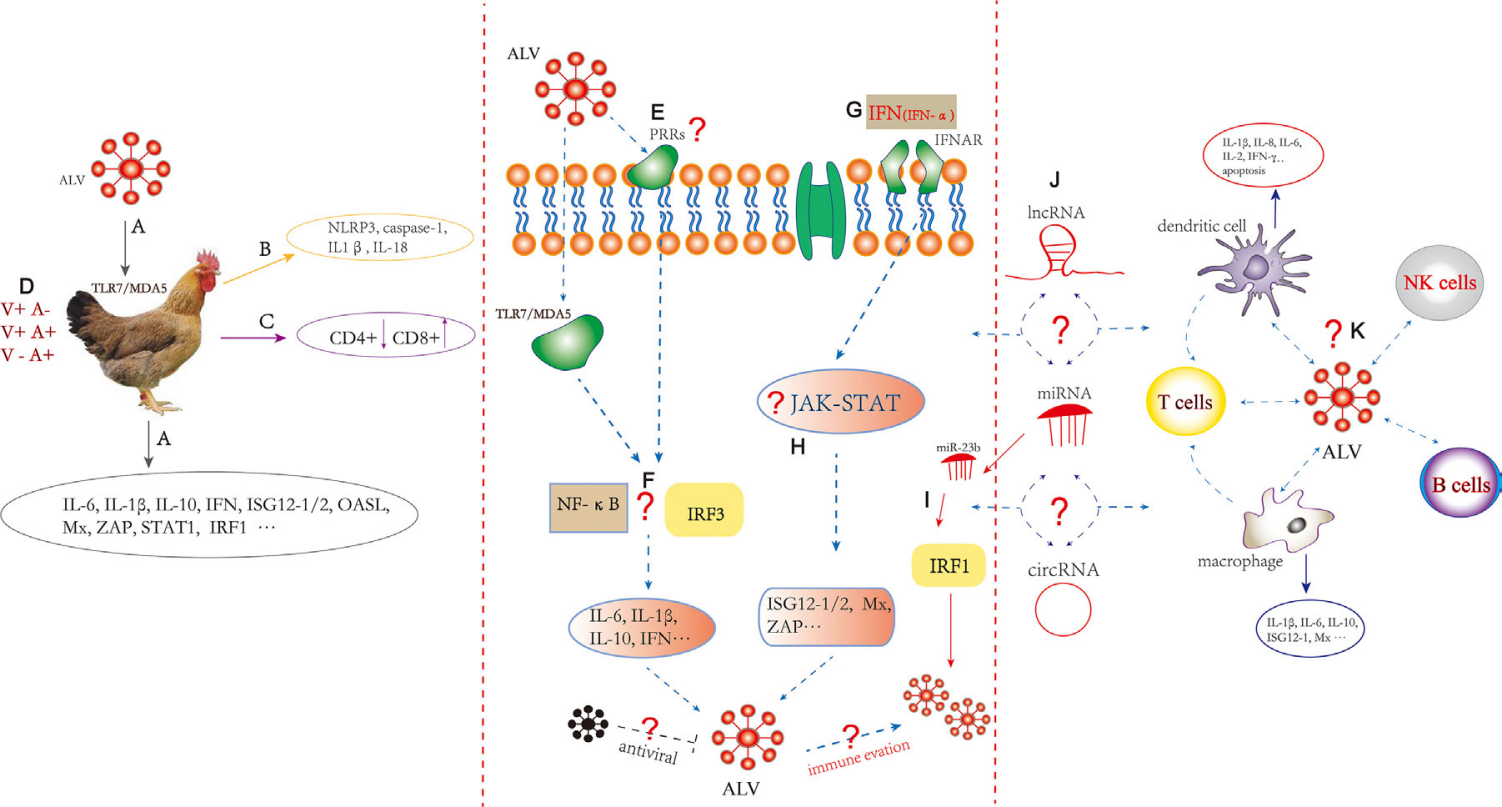
**Innate and adaptive immune responses induced by Avian leukosis virus (ALV)**. **(A)** ALV infection in chickens may be recognized by TLR7 and melanoma differentiation-associated gene 5, followed by induction of innate immunity including differential expression of cytokine and interferon-stimulated genes (ISGs). **(B)** The expression of caspase-1 combined with adaptor NLRP3, IL-1β, and IL-18 increased in ALV-J-infected chick livers. **(C)** CD4^+^ T cell numbers decreased and CD8^+^ T cell numbers increased in the ALV-J-infected chicken spleen. **(D)** Infection with ALV results in three classical *in vivo* infection profiles including (1) V+A− (viremia, no neutralizing antibody); (2) V+A+ (viremia, with neutralizing antibody); and (3) V−A+ (no viremia, with neutralizing antibody). **(E)** The specific pathogen recognition receptors (PRRs) to recognize ALV pathogen-associated molecular patterns should be further studied. **(F)** ALV-A/B/J infection can increase chicken interferon regulatory factors 3 (IRF3) promoter activity in DF-1 cells. Transcription factor such as IRF3 and NF-κB responses to ALV should be further clarified. **(G)** DF-1 cells pretreated with recombinant chicken IFN-α can inhibit the replication of ALV-A/B/J. **(H)** Immune signaling pathway such as PRRs signaling pathway (toll-like receptor, RIG-I-like receptors, interferon-γ-inducible protein 16, and cyclic GMP-AMP synthase) and JAK-STAT signaling pathway responses to ALV should be clarified; the specific mechanism of the inflammatory response, particularly the role of inflammasomes in sensing ALV should be further studied. What immune evasion strategies were used by ALV? Which antiviral factors inhibit the production of ALV? **(I)** miR-23b promotes ALV-J replication by targeting IRF1. **(J)** What is the role of non-coding RNAs including miRNA, long non-coding RNA, and circular RNA in the regulation of innate and adaptive immunity induced by ALV? **(K)** ALV-J can infect chicken dendritic cells (DCs) during the early stages of differentiation and can trigger apoptosis. ALV-J inhibits the differentiation and maturation of DCs and alters cytokine expression that includes IL-1β, IL-8, and IFN-γ. Chicken macrophages are susceptible to ALV-J, and IL-1β, IL-6, ISG12-1, and Mx were altered. The interaction between ALV and macrophages, DCs, natural killer, B cells, CD4^+^, and CD8^+^ T cells needs to be further explored. The dotted line represents remaining processes not fully understood.

Figure 1 summarizes the comprehensive information in ALV immunology area and indicates that our future research should focus on chicken immunity system reacted with ALV. The specific PRRs, ISGs, and the activation of appropriate immune signaling pathways involved with ALV infection should be identified and characterized. The focus should lie on mechanisms of immune evasion and interactions with immune cell including macrophages, DCs, NK, CD4^+^, and CD8^+^ T cells. In addition, the function of endogenous avian leukemia virus in host immunity may be very important as well as interesting (80–82).

In the era of big data, we have access to lots of important information about the host or virus using new technologies such as RNA-Seq. Non-coding RNAs including miRNA, long non-coding RNA, and circular RNA are known to play roles in innate and adaptive immunity (21, 83, 84). Therefore, we also should pay attention to the role of non-coding RNAs in the host immune system with ALV infection.

Above all, we should insist on ALV vaccine development using the basic principles of immunology. Comprehensive understanding of the virus-host interaction would facilitate the development of a successful vaccine.

## Conclusion

Avian leukosis virus-related immunology research is still in infancy. Despite our knowledge of immunosuppression, immunologic tolerance, and antibody response dynamics caused by ALV, there are still major gaps in our understanding of innate immunity and adaptive immunity against these viruses. Based on the limited references, we summarized the comprehensive information in ALV immunology area to assistance for further studies. We hope that the immunity studies of ALV contribute to a further understanding of cancer and AIDS in humans and in the poultry industry itself.

## Author Contributions

MF and XZ drafted the manuscript and approved the final manuscript.

## Conflict of Interest Statement

The authors declare that the research was conducted in the absence of any commercial or financial relationships that could be construed as a potential conflict of interest.
